# Measures of socioeconomic status and self-reported glaucoma in the UK Biobank cohort

**DOI:** 10.1038/eye.2015.157

**Published:** 2015-08-28

**Authors:** Y Shweikh, F Ko, M P Y Chan, P J Patel, Z Muthy, P T Khaw, J Yip, N Strouthidis, P J Foster

**Affiliations:** 1 Division of Genetics and Epidemiology, NIHR Biomedical Research Centre, Moorfields Eye Hospital and UCL Institute of Ophthalmology, London, UK; 2 Department of Public Health and Primary Care, University of Cambridge, Cambridge, UK; 3 Singapore Eye Research Institute, Singapore, Singapore; 4 Discipline of Clinical Ophthalmology and Eye Health, University of Sydney, Sydney, NSW, Australia

## Abstract

**Purpose:**

To determine ocular, demographic, and socioeconomic associations with self-reported glaucoma in the UK Biobank.

**Methods:**

Biobank is a study of UK residents aged 40–69 years registered with the National Health Service. Data were collected on visual acuity, intraocular pressure (IOP), corneal biomechanics, and questionnaire from 112 690 participants. Relationships between ocular, demographic, and socioeconomic variables with reported diagnosis of glaucoma were examined.

**Results:**

In all, 1916 (1.7%) people in UK Biobank reported glaucoma diagnosis. Participants reporting glaucoma were more likely to be older (mean 61.4 *vs* 56.7 years, *P*<0.001) and male (2.1% *vs* 1.4%, *P*=0.001). The rate of reported glaucoma was significantly higher in Black (3.28%, *P*<0.001) and Asian (2.14%, *P*=0.009) participants compared with White participants (1.62%, reference). Cases of reported glaucoma had a higher mean IOP (18 mm Hg both eyes, *P*<0.001), lower corneal hysteresis (9.96 right eye, 9.89 left eye, *P*<0.001), and lower visual acuity (0.09 logMAR right eye, 0.08 logMAR left eye, *P*<0.001) compared with those without (16 mm Hg both eyes, hysteresis 10.67 right eye, 10.63 left eye, 0.03 logMAR right eye, 0.02 logMAR left eye). The mean Townsend deprivation index was −0.72 for those reporting glaucoma and −0.95 for those without (*P*<0.001), indicating greater relative deprivation in those reporting glaucoma. Multivariable logistic regression showed that people in the lowest income group (<£18 000/year) were significantly more likely to report a diagnosis of glaucoma compared with any other income level (*P*<0.01). We observed increasing glaucoma risk across the full range of income categories, with highest risk among those of lowest income, and no evidence of a threshold effect.

**Conclusions:**

In a large UK cohort, individuals reporting glaucoma had more adverse socioeconomic characteristics. Study of the mechanisms explaining these effects may aid our understanding of health inequality and will help inform public health interventions.

## Introduction

Glaucoma is the second commonest cause of blindness worldwide, and the leading cause of medically and surgically irreversible blindness.^1^ In UK, it is the second commonest cause of severe sight impairment registration.^2^ Prevalence of glaucoma increases exponentially with age, with no clear upper limit to the increase in prevalence with age.^3^ As a chronic, age-related disease, current care strategies require regular monitoring of established cases and high-risk suspects. The increase in life expectancy in the UK, together with increasing referral of early cases and suspects has seen 7–8% per year growth in demand for glaucoma service appointments at Moorfields Eye Hospital.^4^ National Institute for Health and Care Excellence (NICE) guidelines for the diagnosis and management of chronic open-angle glaucoma and ocular hypertension in 2009 (NICE, 2009) led to significant increases in referral numbers across the NHS.^5, 6^

Epidemiological data form a core part of the evidence base for clinical decision-making, informing judgements about risk of disease according to age, sex, racial origin, systemic and ocular risk factors. Currently, data on the epidemiology of glaucoma in people of European origin are overwhelmingly derived from studies carried out in the United States, Australia, and the Netherlands.^7, 8, 9, 10, 11^ Prevalence and risk factor studies from the UK at the population level are few and based on work carried out in the 1960s.^12, 13^ More recently, the EPIC Norfolk study^14^ has begun to provide new insights into clinical, anatomical, and genetic characteristics relevant to glaucoma in a predominantly White-UK population.^15, 16, 17^

UK Biobank is a major UK health research resource, which aims to improve the prevention, diagnosis and treatment of a wide range of serious illnesses including cancer, heart diseases, stroke, diabetes, arthritis, osteoporosis, eye disorders, depression and dementia. UK Biobank recruited 502 656 participants aged between 40–69 years in 2006–2010 from 22 centres across the UK. These people have undergone physical measures, provided biological samples, detailed information about themselves and have agreed to have their health status followed. Over many years this will build into a powerful resource to help scientists discover why some people develop particular diseases and others do not.^18^

In 2009, UK Biobank's international advisory committee recommended extending the scope of the study to include health measures, including an eye study module. Baseline vision, refraction, corneal biomechanics, and intraocular pressure (IOP) data are available on 133 668 people, and spectral domain optical coherence tomography and fundus photos are available on 67 321 people.^19^ Linkage to the wealth of health data in UK Biobank make this one the world's most powerful eye and vision research resources.

In this study, we aimed to explore the potential of UK Biobank to study the risk factors and clinical characteristics of glaucoma in the UK, using self-reported diagnosis drawn from baseline questionnaires. We explored links between self-reported glaucoma and ocular, demographic, and socioeconomic factors.

## Materials and methods

The UK Biobank study is a voluntary multisite cross-sectional study of 502 656 UK residents aged 40–69 years who were registered with the National Health Service living within 25 miles from any of the 22 study assessment centres. Nine million people registered with the UK National Health Service were invited to participate via mail, with study response rate of 5.5%. Demographic and socioeconomic details were recorded for all participants. Ethnicity was identified by participants as either White, Chinese, Asian (in this context, typically Indian, Pakistani, or Bangladeshi, not Chinese or other east Asian descent), Black, or Mixed/other. The Townsend deprivation index was used as a measure of deprivation. This Index has been validated for use in a UK-based population^20^, with higher scores representing greater levels of deprivation (range −6.258 to 9.643). Average household annual income before tax was also collected in the questionnaire. Education level and job type were not collected in a readily analyzable format, and thus have not been included in analysis. Self-reported glaucoma was based on those who selected ‘glaucoma' from a predefined list of answers to the question ‘Has a doctor ever told you that you have any of the following problems with your eyes?'

Four years after Biobank study began, additional funding was obtained to collect data on logMAR visual acuity, autorefraction, keratometry, IOP (Goldmann-corrected; Ocular Response Analyzer, Reichert, Depew, NY, USA) and corneal biomechanics at six-study assessment centers among 133 959 eligible participants. Those who did not complete glaucoma question or who did not have valid IOP measurement in at least 1 eye were excluded, leaving a total of 112 690. The North West Multi-centre Research Ethics Committee approved the study (REC Reference Number: 06/MRE08/65), in accordance with the principles of the Declaration of Helsinki. Detailed information about the study is available at the UK Biobank website.^18^

Based on questionnaire responses, we estimated the frequency of self-reported diagnoses of glaucoma in this subset of 112 690 UK Biobank participants. Relationships between demographic, socioeconomic and ocular variables with self-reported diagnoses of glaucoma were examined. Statistical analysis was performed using STATA version 12.0 (Statacorp LP, College Station, TX, USA). Values were considered significant if *P*<0.01.

## Results

**Figure 1:**
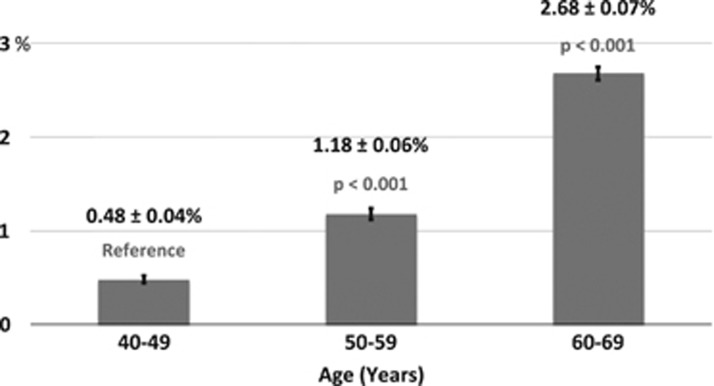
Frequency of self-reported glaucoma by age (%±SE). There is a significantly greater rate of glaucoma reported by older participants (*P*<0.001).

Among 112 690 UK Biobank participants who completed ocular assessments, 1916 (1.7%) reported a diagnosis of glaucoma. Baseline characteristics are reported in Table 1. Mean age for individuals reporting glaucoma was 61.4 years (SE 0.1, SD 6.2) compared with 56.7 (SE 0.2, SD 8.1) years for those without a glaucoma diagnosis (Table 1). The frequency of reported glaucoma significantly increased with older age and male gender (Table 1,Figure 1). Rates of glaucoma were 0.5% among those 40–49 years old, 1.2% among those 50–59 years old, and 2.7% among those 60–69 years old (*P*<0.001 using 40–49 years of age as reference group; Figure 1). A total of 2.1% of men in the study population reported glaucoma compared with 1.4% of women *(P*<0.001).

**Figure 2:**
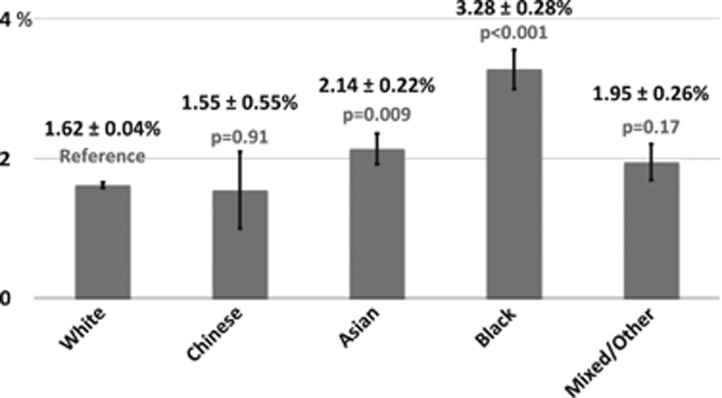
Reported rates of glaucoma by ethnicity (%±SE). The rates of self-reported glaucoma is greatest in Black (*P*<0.001) and Asian (*P*=0.009) participants.

The frequency of self-reported glaucoma was significantly higher amongst Black and Asian participants than the baseline White ethnicity (Figure 2). A total of 3.3% of Black participants (*P*<0.001) and 2.1% of Asian participants (*P*=0.009) reported having glaucoma compared with 1.6% of the White population sampled (Figure 2). There was no significant difference in rates of self-reported glaucoma between Chinese or mixed/other ethnicities as compared with White participants.

Mean Goldmann-corrected (18.26 mm Hg right eye, 18.01 mm Hg left eye) and cornea-corrected IOP (18.87 mm Hg right eye, 18.74 mm Hg left eye) is greater in participants who report a diagnosis of glaucoma (*P*<0.001; Table 1). Both lower corneal hysteresis (9.96 right eye, 9.89 left eye, *P*<0.001) and reduced visual acuity (0.09 logMAR right eye, 0.08 logMAR left eye, *P*<0.001) are significantly associated with self-reported glaucoma (Table 1).

**Figure 3:**
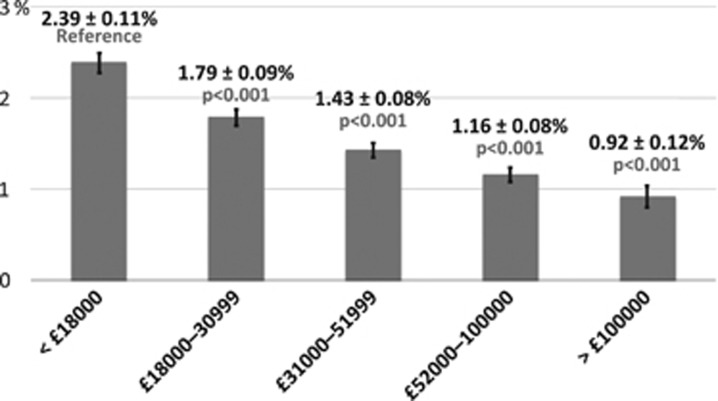
Frequency of self-reported glaucoma by annual income (%±SE). The rate of self-reported glaucoma is inversely related to income, and extends across the full range of the income spectrum.

**Figure 4:**
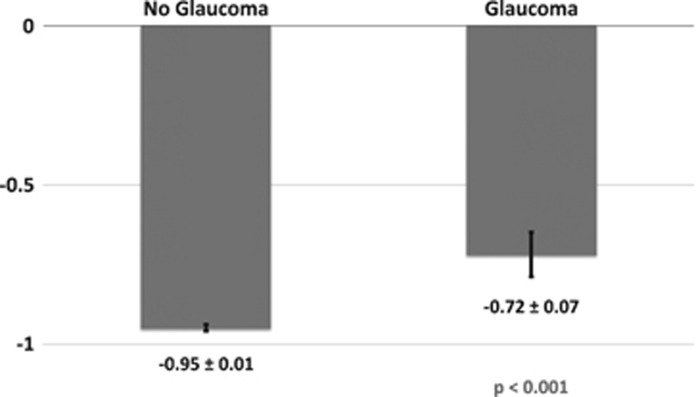
Comparison of Townsend deprivation index between individuals reporting a diagnosis of glaucoma *vs* those who do not (mean Townsend deprivation index±SE). Participants reporting glaucoma were significantly more likely to have a less-negative Townsend deprivation index score (*P*<0.001). This indicates that both groups are less deprived than the UK average (index=0, scores>0 indicate relative deprivation), but those reporting a diagnosis of glaucoma were less likely to be as affluent as those without disease.

As household income decreases, individuals are significantly more likely to report a diagnosis of glaucoma (Figure 3). Rates of glaucoma were highest among those of lowest annual income, <£18 000 (2.4%), and decreased as income increased, with the lowest rates among those with an income of >£100 000/year (0.9%, *P*<0.001 between the highest and lowest groups glaucoma (Table 2)). Chi-squared testing for trend was significant at *P*<0.001. Those who report glaucoma have a higher mean Townsend deprivation index (*P*<0.001; Figure 4). The Townsend deprivation index takes into account factors such as unemployment, non-car ownership, non-home ownership, and household overcrowding.^20^ A more positive score implies a higher degree of deprivation (UK average is score is 0).

Multivariable logistic regression modeling was performed with known risk factors for glaucoma (Table 3). There was a significant association with older age (OR 2.49, 95% CI 1.98–3.12 for ages 50–59 years and OR 5.41, 95% CI 4.37–6.70 for ages 60–69 years, as compared with ages 40–49); male gender (OR 1.50, 95% CI 1.35–1.66); Black or Asian ethnicity (OR 2.81, 95% CI 2.24–3.81 and OR 1.46, 95% CI 1.11-1.91, respectively, compared to Whites); high IOP (OR 1.07 per mm Hg, 95% CI 1.06-1.08) and worse visual acuity (OR 1.08 per 0.1 logMAR, 95% CI 1.06-1.11). Participants with a household annual gross income >£18 000 were statistically less likely to report a diagnosis of glaucoma. Consistent with our findings for income, testing for trends in glaucoma rates by Townsend deprivation index shows a positive correlation, that is, the higher the deprivation index, the more like a participant is to report glaucoma (*P*=0.014, multivariable regression analysis *P*=0.003 for deprivation index ≥1.07).

## Discussion

In this study of a large community cohort of British people, those reporting glaucoma conformed to recognized epidemiological characteristics of those with primary open-angle glaucoma (POAG). Among people of European origin, primary open-angle glaucoma accounts for around 70–85% of all cases of glaucoma, hence risk factors for POAG would be expected to predominate as determinants of disease in a UK population. Self-reported glaucoma cases were older and more likely to be male than those without the diagnosis. The rate of self-reported glaucoma increased nonlinearly with age. Black and Asian people were significantly more likely to report a diagnosis of glaucoma than White participants. Among our study participants, the overall self-reported glaucoma rate was 1.7% in people aged 40–69 years. This rate of disease appears lower than figures from population prevalence studies in European derived populations aged 40 years and older (2.08% Baltimore Whites, USA; 2.0% Melbourne, Australia; 2.9% Egna-Neumarkt, Italy), where participants were all examined for glaucoma^7, 21, 22^ It has been well established in population-based studies in industrialized nations that at least 50% of all glaucoma remains undiagnosed.^10, 23, 24^ One recent study in the US using NHANES data puts the proportion of undiagnosed disease at 78%.^25^ We therefore expect the rate of self-reported glaucoma in UK Biobank to be lower than published population surveys owing to undiagnosed disease.

One of the most striking finding of our study was that, among UK Biobank participants, those who report a diagnosis of glaucoma were more likely to have a lower income and be from a relatively less affluent background. Participants in the lowest income group (<£18 000/year) were significantly more likely to have self-reported glaucoma, compared with any other income category, after adjusting for age, sex, race, IOP, and visual acuity. The mean Townsend deprivation index was −0.72 for those reporting glaucoma and −0.95 for those without (*P*<0.001). This indicates that both groups are less deprived than the UK average (index=0, scores>0 indicate relative deprivation), but those reporting a diagnosis of glaucoma were less likely to be as affluent as those without disease. Furthermore, we observed a gradient toward higher rates of glaucoma with decreasing income across the socioeconomic spectrum. Among a cohort who are less deprived than the UK average, the findings of reduced affluence among those reporting disease than in those who do not, and of a trend toward increased rate of disease across the spectrum of income groups are both novel, and important for understanding the mechanisms which drive the previously reported association between socioeconomic deprivation and late presentation of glaucoma.

Previous research has established that higher rates of glaucoma are seen in those at the lowest end of the socioeconomic spectrum, and that these people tend to present later, with more advanced disease, primarily owing to limited access to primary eye care services. Higher rates of glaucoma have been observed in both homeless and poor nonhomeless populations than in the general population of Los Angeles.^26^ In the UK, greater individual and area level deprivation are associated with late presentation of glaucoma.^27, 28, 29^ People presenting with advanced glaucoma are more likely to come from an underprivileged area and be of lower occupational class, to have no access to a car, to have left full time education at age 14 years or younger, and to be tenants rather than owner occupiers.^27^

The theory that later presentation is related to deprivation is apparently supported and explained by findings from the UK and Canada. A geographical mapping study in the UK identified a clear disparity between areas of deprivation and location of optometric services.^28^ In Canada, patients with newly diagnosed glaucoma were less likely to come from the poorest neighborhood areas (16%, compared with an expected 20%, *P*=0.56). Compared with those from the poorest areas, people from the richest neighborhoods appeared to have a substantially lower risk for having moderate or advanced glaucoma at first presentation (prevalence ratio 0.66, 95% confidence interval: 0.43–1.02, *P*=0.06). This association was stronger among those ≥65 years old (*P*=0.006). These findings may suggest that socioeconomic deprivation is associated with greater severity of glaucoma at presentation, and may indicate relative underdetection of glaucoma in poorer groups.^30^ Fraser *et al*^27^ also speculated that material deprivation may be associated with ‘more aggressive' disease, as well as later presentation. Our findings may offer some support for this theory. There do not appear to be two groups (the ‘deprived' and the ‘wealthy') and certainly there does not appear to be a clear threshold at which the impact of deprivation is felt. The same pattern of a spectrum of increasing risk has been seen for all cause mortality, coronary heart disease mortality, and all cause vascular mortality across the range of deprivation (index of multiple deprivation) in a longitudinal study of British women. The authors commented that these relationships seemed to be mediated largely, although not exclusively, through health-related behavior such as physical activity, alcohol consumption, fruit and vegetable intake, and smoking.^31^

There is a clear relationship between deprivation and access to eye care services in both developing and developed world settings. In Pakistan, cataract surgical coverage was higher in affluent clusters (80.6%) than in medium (76.8%) and poor areas (75.1%). Intraocular lens implantation rates were significantly lower in participants from poorer households. 10.2% of adults living in affluent clusters presented to the examination station wearing spectacles, compared with 6.7% in medium clusters and 4.4% in poor cluster areas. Spectacle coverage in affluent areas was more than double that in poor clusters (23.5% *vs* 11.1%, *P*<0.001).^32^ In a cross-sectional sample representing the non-institutionalized US population aged 40+, people with a low poverty-income ratio and an age-related eye disease were less likely to have visited an eye care provider (62.7% *vs* 80.1% *P*<0.001) or undergone a dilated eye examination in the past 12 months (64.3% *vs* 80.4% *P*<0.001), compared with people at the higher end of the income spectrum, after adjustment for other factors. Similarly, persons with less than a high school education were less likely than those with at least a college education to report a visit to an eye care provider (62.9% *vs* 80.8% *P*<0.001) or to have had a dilated eye examination (64.8% *vs* 81.4% *P*<0.001).^33^ In addition, it has been shown that among patients presenting with a fractured neck of femur, binocular visual acuity is <6/12 in 46%.^34^ Those who were visually impaired were more likely to have symptomatic visual complaints (58% *vs* 26%), but were less likely to be under optometric care (71% *vs* 85%).^34^ A higher proportion of the group with visual impairment lived in areas of social deprivation (40% *vs* 26%).^34^ The majority of cases in this group of individuals are not in touch with ophthalmic services, leading the authors to comment that social deprivation appeared to be associated with the inability to access ophthalmic care.^34^

In the UK, the national healthcare system provides the majority of care at little or no charge. However, barriers to accessing regular eye examinations may still exist, including poor knowledge of eye health, concerns about the cost of spectacles, mistrust of optometrists and limited geographical access in socioeconomically deprived areas. These are believed to result in low uptake of services, and subsequent late presentation to ophthalmology clinics.^35^ Day *et al*^28^ suggested that it may not be feasible to rely on private ‘high street' optometrists to provide primary eye care services for the most deprived. There have been calls for noncommercial primary eye care in the form of ophthalmic or optometric community services to provide primary eye care in socioeconomically deprived areas. Funding would be required from sources other than eye examination fees and spectacle sales. In order to promote greater access to care, there have been calls for a significant shift of activity from secondary to primary care locations. It has been suggested that costs incurred could be offset by the utility gain from earlier detection of preventable sight loss.^35^

Limitations of this study clearly include the use of self-reported disease status, which can result in misclassification error. Participants with ocular hypertension or suspect glaucoma may mistakenly report a diagnosis of glaucoma. There will be under-ascertainment of people with disease, as around 50% of all cases will not have been diagnosed and therefore will not be captured. The overall direction of impact of these potential errors is unknown. However, we believe the overall pattern seen in the data would not be diminished. On the contrary, if people from more deprived background have greater barriers to health seeking behavior and care, one would expect that they would be less likely to be aware of their diagnosis and not report it, and probably less likely to attend health research activities such as UK Biobank. We believe it is likely the true trends identified here for socioeconomics influencing the risk of glaucoma would be accentuated, if the true burden of disease were identified. We were not able to differentiate between POAG, angle-closure glaucoma and secondary disease, and hence are not able to comment on the relative impact of socioeconomics on different forms of glaucoma. It has been reported that acute primary angle-closure risk is higher in people from deprived backgrounds.^36^ Finally, as UK Biobank has a low response rate of 5.5%, figures for rates of disease must be treated with caution. The low participation rate limits external validity and is not representative of the UK population. However, the study size of 112 690 is a major strength that allows us to detect and quantify small effects. We also used standardized techniques, and included individuals from several different ethnicities.

In the UK Biobank cohort, the distribution of self-reported glaucoma shows expected associations with higher rates of disease in the elderly, men, and Black and Asian people. As a novel finding, we have identified an inverse relationship between self-reported glaucoma and measures of socioeconomic status. The cohort is of a higher socioeconomic status than the average members of the UK population. The relationship between income category and rate of self-reported glaucoma showed a clear trend of increasing disease across the full spectrum of income. These two facts give an important new perspective to views on the relationship between glaucoma and socioeconomics. There are undoubtedly barriers to eye care for detection and treatment. However, studies of mortality point towards the effects of deprivation being mediated through ‘health-related behaviors' such as physical activity, alcohol consumption, fruit and vegetable intake, and smoking.

We now plan to examine structural biomarkers for glaucoma in the UK Biobank cohort (such as retinal nerve fiber layer thickness on OCT examinations) and assess firstly if the socioeconomic relationship exists with evidence of end organ damage, and secondly to use data soon to become available, such as the serum biomarker panel, diet, and physical exercise data, on this cohort to try to determine the biological mechanisms, which underlie the novel findings reported here.


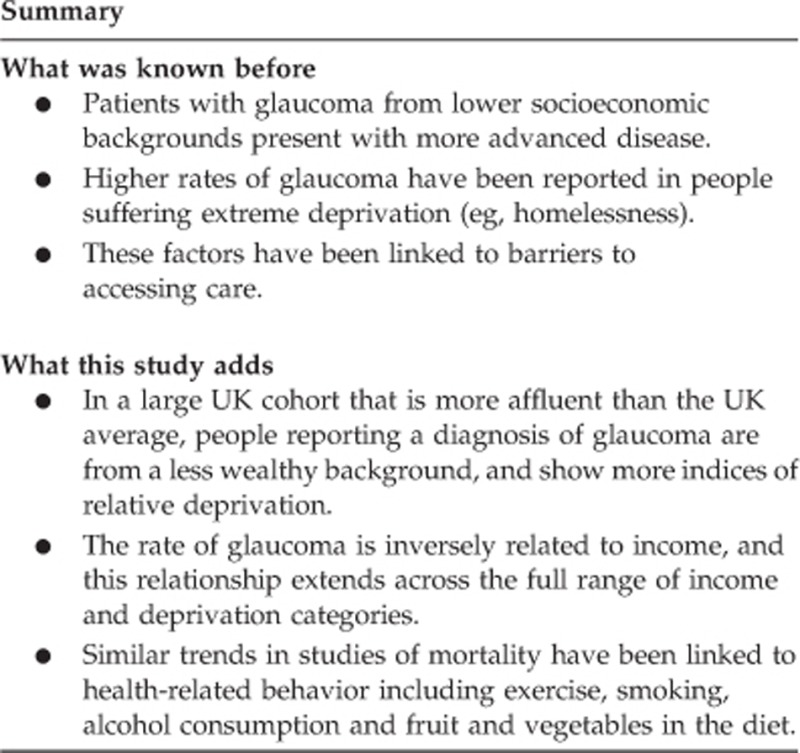


## Figures and Tables

**Table 1:** Participant characteristics

	*No glaucoma*	*Glaucoma*
*Age	56.7±0.02	61.4±0.14
^+^Male gender	45.6±0.1%	56.6±1.1%
		
^*+*^*Ethnicity*		
White	89.7±0.1%	85.1±0.8%
Chinese	0.5±0.02%	0.4±0.1%
Asian	3.8±0.1%	4.8±0.5%
Black	3.4±0.1%	6.8±0.6%
Mixed/Other	2.5±0.04%	2.8±0.4%
		
^*+*^*Income*		
<£18000	21.7±0.1%	31.9±1.2%
£18000–30999	24.9±0.1%	27.2±1.1%
£31000–51999	25.7±0.1%	22.3±1.0%
£52000–100000	21.1±0.1%	14.9±0.9%
>£100 000	6.5±0.1%	3.6±0.5%
*Townsend deprivation index	−0.95±0.01	−0.72±0.07

*Mean or ^+^percentage±SE.

**Table 2:** Multivariable regression analysis of risk factors for reported glaucoma

	*Odds ratio*	*95% confidence interval*		P*-value*
*Age, years*				
40–49	Reference	Reference		Reference
50–59	2.49	1.98	3.12	<0.001
60–69	5.41	4.37	6.70	<0.001
Male gender (*vs* female)	1.50	1.35	1.66	<0.001
				
*Ethnicity*				
White	Reference	Reference		Reference
Chinese	1.17	0.50	2.73	0.72
Asian	1.46	1.11	1.91	0.006
Black	2.81	2.24	3.51	<0.001
Mixed/other	1.45	1.02	2.06	0.04
				
*Income*				
<£18 000	Reference	Reference		Reference
£18 000–30 999	0.82	0.71	0.94	0.003
£31 000–51 999	0.82	0.68	0.91	0.001
£52 000–100 000	0.78	0.66	0.92	0.003
>£100 000	0.64	0.48	0.86	0.003
IOPcc	1.07	1.06	1.08	<0.001
Visual acuity (per 0.1 logMar)	1.08	1.06	1.11	<0.001

Abbreviation: IOPcc, cornea-corrected intraocular pressure in right eye.

Visual acuity from right eye.

**Table 3:** Comparison of IOP, corneal biomechanics and visual acuity between participants reporting glaucoma and those who did not (mean±SE)

	*No glaucoma*	*Glaucoma*	P-*value*
*IOP (mm Hg, Goldmann-corrected)*			
Right	15.84±0.01	18.26±0.12	<0.001
Left	15.70±0.01	18.01±0.12	<0.001
			
*IOP (mm Hg, cornea-corrected)*			
Right	16.02±0.01	18.87±0.12	<0.001
Left	15.95±0.01	18.74±0.13	<0.001
			
*Corneal hysteresis*			
Right	10.67±0.01	9.96±0.07	<0.001
Left	10.63±0.01	9.89±0.06	<0.001
			
*Corneal resistance*			
Right	10.74±0.01	10.86±0.07	0.03
Left	10.66±0.01	10.72±0.06	0.30
			
*Visual acuity (logMar)*			
Right	0.025±0.001	0.088±0.005	<0.001
Left	0.021±0.001	0.083±0.006	<0.001

A self-reported diagnosis of glaucoma was significantly associated with greater IOP (*P*<0.001), reduced corneal hysteresis (*P*<0.001) and reduced visual acuity (*P*<0.001).
